# Contemporary Trends in Hospitalizations for Comorbid Chronic Liver Disease and Substance Use Disorders

**DOI:** 10.14309/ctg.0000000000000372

**Published:** 2021-06-18

**Authors:** Archita P. Desai, Marion Greene, Lauren D. Nephew, Eric S. Orman, Marwan Ghabril, Naga Chalasani, Nir Menachemi

**Affiliations:** 1 Department of Gastroenterology and Hepatology, School of Medicine, Indiana University, Indianapolis, Indiana, USA;; 2 Department of Health Policy and Management, Richard M. Fairbanks School of Public Health, Indiana University, Indianapolis, Indiana, USA.

## Abstract

**INTRODUCTION:**

Chronic liver diseases (CLDs) and substance use disorders (SUDs) are increasingly prevalent and often coexist. Contemporary studies describing the characteristics and hospitalization trends of those with comorbid CLD-SUD are lacking. We aimed to characterize a population-based cohort with comorbid CLD-SUD and describe trends in these hospitalizations over time by individual-level characteristics.

**METHODS:**

We performed a cross-sectional analysis of the National Inpatient Sample from 2005 through 2017. Diagnosis codes were used to identify adult hospitalizations with CLD, SUD, or both. Bivariate and multivariate analyses were used to make comparisons between diagnosis categories. Unadjusted and age-adjusted trends in these hospitalizations were described over time.

**RESULTS:**

Of 401,867,749 adult hospital discharges, 3.2% had CLD-only and 1.7% had comorbid CLD-SUD. Compared with CLD-only, comorbid CLD-SUD hospitalizations resulted in higher inpatient mortality (3.1% vs 2.4%, *P* < 0.001) and were associated with younger age, male sex, Native American race, and urban and Western US location. Over time, comorbid hospitalizations grew 34%, and the demographics shifted with larger increases in hospitalization rates seen in younger individuals, women, Native Americans, and those publicly insured. In comorbid hospitalizations, alcoholic SUD and CLD decreased, but drug SUDs and nonalcoholic fatty liver diseases are fast-growing contributors.

**DISCUSSION:**

In this comprehensive analysis of US hospitalizations, comorbid CLD-SUD hospitalizations are increasing over time and lead to higher inpatient mortality than CLD alone. We further characterize the changing demographics of these hospitalizations, providing a contemporary yet inclusive look at comorbid CLD-SUD hospitalizations. These data can guide interventions needed to improve the poor outcomes suffered by this growing population.

## INTRODUCTION

Chronic liver diseases (CLDs) and substance use disorders (SUDs) frequently coexist, and their occurrence is each increasing in the United States. Over the past decade, the prevalence of CLD has increased and is matched by increasing CLD-related mortality, currently estimated at 14.2 persons per 100,000 (1,2). Rates of SUD are also rising with national estimates showing that 7.8% of US adults were inflicted in the past year (3). Furthermore, overdose mortality because of accidental drug or alcohol poisoning nearly doubled in the past 10 years and is currently at 18.9 persons per 100,000 (4). Therefore, comorbid CLD and SUD is common and presents a potentially growing challenge, yet studies describing this vulnerable patient population are lacking (5).

Studies focusing on CLD highlight important differences in trends of hospitalizations, healthcare costs, and mortality by age group, race, and ethnicity (6–10). Specifically, CLD because of SUDs, such as alcoholic liver disease and injection drug use-related hepatitis C virus (HCV), is prevalent and increasing in young individuals (11–18). CLD-related mortality has increased in Whites but disproportionately increased in American Indians and Alaskan Natives leading to significant increases in premature mortality (17–21). Previous studies have also identified regional variations in the outcomes of hospitalizations for CLD across the United States (17,21–23). However, most studies have not extended their analyses to the period after 2014 when curative therapies for HCV were introduced (12,14,16,18–24). Existing studies using more recent national data are limited in that they either have focused on alcohol (11,13,15) or have been limited to end-stage liver disease (17). Therefore, to date, no study has described the contemporary characteristics of hospitalized patients with all-cause CLD and SUD over time.

Given this gap in our knowledge, we aimed to study the characteristics of a population-based cohort with CLD and SUD and to describe hospitalization trends over time because of SUD and CLD by age group, race, and ethnicity. The inpatient setting is a common source of healthcare use for those with CLD (1,9), and with the growing epidemic of both CLD and SUD, understanding the characteristics of individuals hospitalized with CLD and SUD can better inform public policymaker and the frontline providers who commonly see these patients (i.e., mental health providers, gastroenterologists, and hepatologists).

## METHODS

### Research design and data source

This study is a repeated cross-sectional analysis of the National Inpatient Sample (NIS) from 2005 through 2017 (25). The NIS is an all-payer inpatient database covering 97% of the US population and representing more than 35 million annual hospitalizations. Further details on the NIS survey design are available online (25). For all years, each individual hospitalization is deidentified and carries demographic details, including age, sex, race/ethnicity, insurance provider, and zip code-based income quartile. The NIS also includes hospital characteristics and discharge status. We defined SUD and CLD hospitalizations based on the presence of *International Classification of Diseases* (ICD), 9th or 10th revision diagnosis and procedure codes used in previous literature (6,7,26,27) (*International Classification of Diseases*, 10th revision since fourth quarter 2015; see Supplementary Table 1, Supplementary Digital Content 1, http://links.lww.com/CTG/A638). This study was exempt from human subjects research by the Indiana University Human Research Protection Program, as defined in 45 CFR 46.102(f).

### Inclusion criteria

All adult hospital discharges from 2005 to 2017 were assessed for inclusion. We considered the first 15 diagnosis fields to identify records with CLD, SUD, and comorbid CLD-SUD. Hospitalizations were included in the CLD-only group if they contained diagnosis codes for CLD but not for SUD. Similarly, SUD-only hospitalizations had diagnosis codes for SUD but not for CLD. Finally, the comorbid CLD-SUD hospitalizations contained diagnosis codes for both conditions. All other hospitalizations were considered as a fourth group.

### Statistical analysis

Bivariate analyses were used to describe the study population based on their diagnosis category during hospitalization. For all groups, we examined differences in patient characteristics (sex, age, race/ethnicity, insurance, and median income quartile-based on zip code) and hospital characteristics (rural/urban and US census region). We then conducted multivariable logistic regression analyses to identify the factors associated with having comorbid CLD-SUD (dependent variable) among hospitalizations with a CLD diagnosis. We included patient and hospital characteristics as independent variables and adjusted for temporal trends using dummy variables for each year to model the nonlinear trends we observed. We also measured unadjusted trends in hospitalization and age-adjusted trends in inpatient mortality and length of stay (LOS) over time for each of the aforementioned diagnosis categories. Next, we reviewed trends of the major CLD and SUD types. Finally, we analyzed trends by sex, age, race/ethnicity, insurance, and region in comorbid CLD-SUD. Whenever appropriate, odds ratios and 95% Wald confidence intervals were computed. Given the large sample size, many relationships are statistically significant. Consistent with other studies, we highlight the ones with *P* < 0.001. All analyses were conducted using Statistical Analysis Software version 9.4 (SAS Institute, Cary, NC).

## RESULTS

There were 401,867,749 total adult hospital discharges from 2005 to 2017. Of these, 12,791,036 (3.2%) had CLD-only, 28,579,878 (7.1%) had SUD-only, and 6,929,801 (1.7%) had comorbid CLD-SUD (Table 1). Discharges with comorbid CLD-SUD had an average age of 49.2 years, 31.5% were female, and 81.0% had public insurance. Liver disease was due to alcohol (49.8%), HCV (46.3%), and cirrhosis (39.4%); 74% had alcohol use disorder, and 42.5% had a drug use disorder.

**Table 1.** Patient and hospital characteristics by diagnosis group

Patient and hospital characteristics	CLD-only (N = 12,791,036 [3.2%])	SUD only (N = 28,579,878 [7.1%])	Comorbid CLD-SUD (N = 6,929,801 [1.7%])	Other hospitalizations (N = 353,567,034 [88.0%])
Mean age, yr (SD)^a^	58.8 (33.7)	46.3 (35.6)	49.2 (26.7)	58.2 (46.4)
Age group^a^				
18–35	7.9%	29.2%	13.9%	20.7%
36–50	19.1%	30.5%	34.2%	14.5%
51–64	37.9%	26.6%	42.0%	20.3%
65 and older	35.2%	13.7%	10.0%	44.6%
Female^a^	49.8%	37.4%	31.5%	62.2%
Race^a^				
White	64.1%	64.8%	65.3%	69.2%
Black	15.6%	21.5%	15.8%	13.8%
Hispanic	12.9%	9.0%	13.7%	10.8%
Asian or Pacific Islander	3.5%	0.9%	0.8%	2.6%
Native American	0.8%	1.0%	1.5%	0.6%
Other	3.1%	2.8%	3.0%	3.0%
Median income by patient zip code^a^				
Quartile 1 (lowest income)	32.3%	36.0%	37.1%	28.8%
Quartile 2	25.7%	25.5%	25.4%	26.0%
Quartile 3	23.0%	21.7%	21.6%	24.0%
Quartile 4 (highest income)	21.2%	19.0%	16.8%	16.0%
Expected primary payer^a^				
Public insurance, self-pay, or other	73.8%	77.3%	81.0%	69.5%
Private insurance	26.2%	22.7%	19.0%	30.5%
Hospital location^a^				
Rural	8.6%	9.6%	8.1%	11.9%
Urban	91.4	90.4%	91.9%	88.1%
Hospital region^a^				
Northeast	18.7%	22.4%	22.9%	19.2%
Midwest	19.6%	23.3%	19.0%	23.1%
South	39.8%	34.5%	34.5%	39.1%
West	21.9%	19.8%	23.5%	18.7%
Liver disease etiology^b^				
Alcohol	6.9%	—	49.8%	—
Hepatitis C	31.3%	—	46.3%	—
NAFLD	28.3%	—	7.0%	—
Cirrhosis	31.1%	—	39.4%	—
Type of SUD				
Alcohol use disorder	—	54.8%	73.7%	—
Drug use disorder	—	60.7%	42.5%	—
Length of stay				
Mean (SD)^a^	6.1 (17.8)	5.2 (15.5)	5.6 (15.4)	4.6 (13.5)
Median (Q1–Q3)	4.0 (2.0–7.0)	3.0 (2.0–6.0)	4.0 (2.0–7.0)	3.0 (2.0–5.0)
Died^a^				
Unadjusted	6.4%	0.9%	3.0%	2.2%
Age-adjusted	1.9%	2.4%	3.1%	1.5%

CLD, chronic liver disease; NAFLD, nonalcoholic fatty liver disease; SUD, substance use disorder.

a *P* < 0.0001.

b Categories not mutually exclusive.

**Figure 1.**
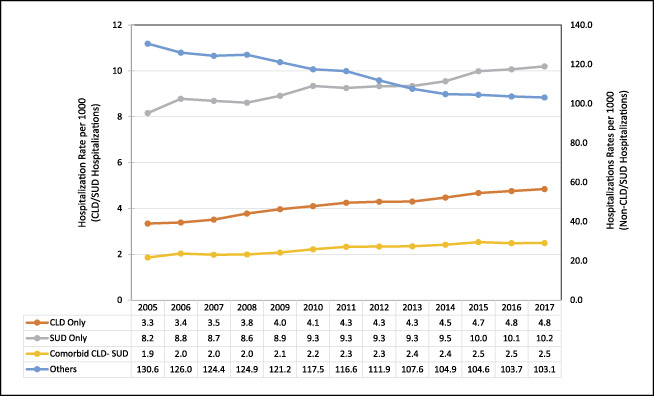
Trends in hospitalization rate per 1,000 populations by year and by diagnosis group over the study period. CLD, chronic liver disease; SUD, substance use disorder.

Hospitalization rates per 1,000 persons from 2005 to 2017 are presented in Figure 1. Whereas per capita hospitalizations have decreased overall, and hospitalizations for CLD, SUD, and comorbid CLD-SUD increased during the study period. From 2005 to 2017, comorbid CLD-SUD hospitalizations increased by 34% and CLD-only hospitalizations increased by 45%.

### Factors associated with comorbid CLD-SUD hospitalizations

We observed variation in demographic and geographic characteristics across the groups (Table 1). Those with comorbid CLD-SUD were younger than those with CLD-only but older than those with SUD-only (49.2 vs 58.8 vs 46.3 years, respectively). In addition, those with comorbid CLD-SUD were less likely to be female compared with either the CLD- or SUD-only groups (31.5% vs 49.8% vs 37.4%, respectively). Discharges with comorbid CLD-SUD were also more likely to report Hispanic ethnicity (13.7% vs 12.9% CLD-only vs 9.0% SUD-only), live in the lowest income zip code (37.1% vs 32.3% CLD-only vs 36.0% SUD-only), and have public insurance (81.0% vs 73.8% CLD-only vs 77.3% SUD-only).

Compared with the CLD-only group, those with comorbid CLD-SUD were more likely to have alcohol-related and HCV-related liver disease and less likely to have nonalcoholic fatty liver disease (NAFLD) (Table 1). The comorbid group was also more likely to have cirrhosis compared with the CLD-only group (39.4% vs 31.1%). The type of SUD also varied in those with comorbid CLD-SUD compared with the SUD-only group with higher rates of alcohol use disorder (69.0% vs 48.7%; see Supplementary Table 2, Supplementary Digital Content 1, http://links.lww.com/CTG/A638), but lower rates of cannabis use disorders (11.3% vs 26.5%).

After adjusting for demographic, geographic, and temporal variables, comorbid CLD-SUD hospitalizations were negatively associated with increasing age (adjusted odds ratio [aOR] 0.97) and black, Hispanic, and Asian race/ethnicity (aOR 0.80, 0.85, 0.85) compared with CLD-only (Table 2). On the other hand, men (aOR 3.74), Native Americans (aOR 1.75), and individuals with public insurance (aOR 2.14) were each associated with increased odds of comorbid CLD-SUD. In addition, those living in the lowest income zip code (aOR 1.50) and the Western US region (aOR 1.07) were more represented among comorbid CLD-SUD hospitalizations, whereas rural location was less represented (aOR 0.68). Odds of comorbid CLD-SUD hospitalizations increased annually from 2005 to 2017, as compared with CLD-only.

**Table 2.** Odds of having comorbid CLD-SUD within CLD hospitalizations

Variable	OR	95% CI
Age	0.97	0.97–0.97
Sex (ref = female)		
Male	3.74	3.73–3.74
Race (ref = white)		
Asian/Pacific Islander	0.30	0.30–0.31
Black	0.80	0.79–0.80
Hispanic	0.85	0.85–0.86
Native American	1.75	1.74–1.76
Others	0.72	0.72–0.72
Payer (ref = private insurance)		
Public, self-pay, or other	2.14	2.14–2.15
Median income by patient zip code (ref = 76th–100th percentile)		
0–25th percentile	1.50	1.49–1.50
26th–50th percentile (median)	1.21	1.21–1.22
51st–75th percentile	1.13	1.12–1.13
Hospital urbanicity (ref = urban)		
Rural	0.68	0.68–0.68
Hospital region (ref = Northeast)		
Midwest	0.72	0.72–0.73
South	0.73	0.73–0.74
West	1.07	1.07–1.07
Year (ref = 2005)		
2006	1.11	1.11–1.12
2007	1.08	1.07–1.08
2008	1.08	1.07–1.08
2009	1.09	1.09–1.10
2010	1.18	1.17–1.19
2011	1.29	1.28–1.29
2012	1.31	1.30–1.32
2013	1.37	1.36–1.38
2014	1.42	1.41–1.43
2015	1.49	1.48–1.50
2016	1.46	1.45–1.47
2017	1.48	1.47–1.48

CI, confidence interval; CLD, chronic liver disease; OR, odds ratio; SUD, substance use disorder.

### Outcomes in comorbid CLD-SUD hospitalizations

**Figure 2.**
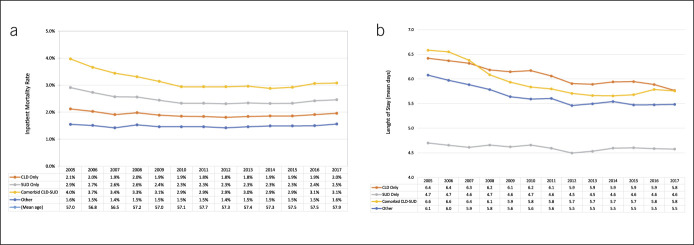
Trends in outcomes by year and by diagnosis group over the study period. (**a**) Trends in age-adjusted in-hospital mortality. (**b**) Trends in mean, age-adjusted length of stay. CLD, chronic liver disease; SUD, substance use disorder.

Those with CLD-only had the highest rate of unadjusted in-hospital mortality (6.4%) followed by comorbid CLD-SUD (3.0%, Table 1). Once adjusted for age, inpatient mortality was highest for CLD-SUD (3.1%) followed by the SUD-only group (2.4%) and the CLD-only group (1.9%) compared with all other hospitalizations (1.5%, Figure 2a). Age-adjusted inpatient mortality for the CLD-only group remained relatively unchanged over the study period, ranging from 1.8% to 2.1%. In those with comorbid CLD-SUD, however, age-adjusted mortality decreased early in the study period from 4.0% to 2.9% in 2010 and remained around this rate until 2016 when it increased slightly to 3.1% (Figure 2a). The median LOS for the comorbid group was 4.0 days, similar to that of the CLD-only group (Table 1). Overall, LOS for both groups decreased until 2010. After this time, it has remained unchanged for the CLD-SUD group while continuing to slowly decrease in the CLD-only group (Figure 2B).

### Trends in hospitalization features in comorbid CLD-SUD

**Figure 3.**
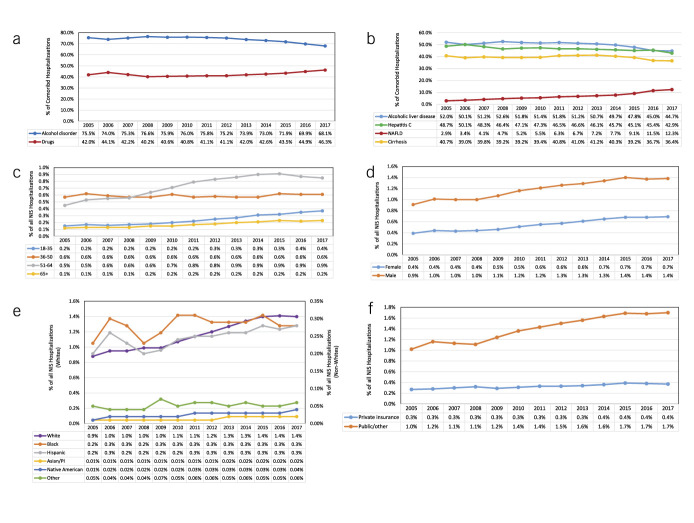
Trends in comorbid CLD-SUD hospitalizations by year and by relevant clinical or demographic characteristics. (**a**) Percent of comorbid hospitalizations with alcohol and drug use disorders over the study period. (**b**) Percent of comorbid hospitalizations with alcoholic liver disease, HCV, NAFLD, and cirrhosis over the study period. (**c**) Percent of all adult NIS hospitalizations with comorbid CLD-SUD by age group. (**d**) Percent of all adult NIS hospitalizations with comorbid CLD-SUD by sex. (**e**) Percent of all adult NIS hospitalizations with comorbid CLD-SUD by race/ethnicity. (**f**) Percent of all adult NIS hospitalizations with comorbid CLD-SUD by expected payor. CLD, chronic liver disease; HCV, hepatitis C virus; NAFLD, nonalcoholic fatty liver disease; SUD, substance use disorder.

Figure 3 shows important trends in the characteristics of comorbid CLD-SUD hospitalization. Although alcohol use disorder decreased over time, it remained a major diagnosis group in comorbid hospitalizations. Other drug use disorders have grown by 10% since 2014 (Figure 3a). This is similar to the trends seen in the SUD-only group (see Supplementary Figure 1A, Supplementary Digital Content 1, http://links.lww.com/CTG/A638). The type of CLD is also shifting over time (Figure 3b; see Supplementary Figure 1B, Supplementary Digital Content 1, http://links.lww.com/CTG/A638). Hepatitis C decreased in both groups, but less decrease was seen in the comorbid group (12%) vs the CLD-only group (34%). By contrast, NAFLD has significantly grown in both groups with a larger increase in the CLD-SUD group (320%) vs the CLD-only group (74%). Finally, cirrhosis rates have slightly decreased in the CLD-SUD group, whereas remaining relatively stable in the CLD-only group.

Figure 3 also depicts trends within hospitalizations with comorbid CLD-SUD by individual characteristics. CLD-SUD hospitalizations in all age groups grew except for those aged 36–50 years, with the largest increase in the 18–35 age group (Figure 3c). In addition, the rate of female comorbid CLD-SUD hospitalization increased more than the rate of males (Figure 3d). An increase in comorbid CLD-SUD hospitalizations varied by the racial/ethnic group over the study period (Figure 3e). Comorbid hospitalizations among Native Americans increased by 300% while increasing at smaller magnitudes among blacks, Hispanics, whites, and Asian/Pacific Islanders (22% vs 40% vs 59% vs 100%, respectively). CLD-SUD hospitalizations covered by public insurance increased by 67%, whereas those covered by private insurance grew only by 37% (Figure 3f).

## DISCUSSION

In this comprehensive study spanning over a decade of US hospitalizations, we provide a contemporary examination of trends among individuals admitted with both CLD and SUD. We find that hospitalizations because of either condition have grown steadily. Compared with CLD-only hospitalizations, comorbid hospitalizations resulted in higher inpatient mortality and were concentrated among younger individuals, men, Native Americans, individuals in lower income zip codes, those publicly insured, and those living in rural areas and in Western states. Importantly, over time, the demographics of comorbid hospitalizations have changed to include a greater growth in women, young adults, Native Americans, and those with public insurance.

The epidemiology of alcoholic CLD has been the subject of recent studies, which establish that alcoholic CLD has increased in the United States (11–14,17). However, our study is the first to report rates of comorbid SUD without limitation to the type of CLD. Although hepatitis C-related liver disease hospitalization rates have remained steady, hospital rates for NAFLD are increasing greatly and disproportionately for those with comorbid CLD-SUD (320% vs 74% in CLD-only). Therefore, it is important to understand the impact of concomitant SUD for this growing population. Previous research shows that obesity and its complications are more prevalent in those with SUD because of diet and lifestyle, adverse effects of psychotropic medications, and higher prevalence of poverty (28). The presence of NAFLD also has the potential to affect outcomes related to SUD through altered pharmacokinetics and drug clearance (29,30). Alcohol remains an important SUD, and recent studies have shown increased mortality and rates of liver cancer in those with NAFLD who engage in even modest alcohol consumption, possibly due to shared pathophysiology, molecular pathways, and genetic risk factors leading to steatohepatitis and subsequent liver fibrosis (31–33). Taken together with growing rates of NAFLD in comorbid CLD-SUD in all hospitalizations, future studies investigating shared mechanisms of CLD and SUD disease and integrated treatment are warranted.

In addition to shared pathophysiology, CLD and SUD share common demographic traits which increase the risk of coexistence. In a previous study using the NIS, SUD hospitalization rates were higher in those with alcoholic CLD; however, this study excluded those with alcohol use disorder and did not look at trends over time (16). In our analysis, as hospitalization rates for CLD and SUD increase over time, the burden of comorbid CLD-SUD has persisted, making up 15% of all CLD or SUD admissions. This is likely due to national trends in CLD showing younger, female, white and Native American individuals experiencing disproportionately increasing rates of CLD and SUD (1,11,12,17,18). In our study, these same demographic traits are more likely to be seen in the comorbid CLD-SUD population.

For example, the groups within the comorbid CLD-SUD cohort that were observed to have the greatest growth over time included the youngest (age 18–35 years) and oldest (51+ years). Several studies have shown increasing rates of CLD in younger individuals and the baby boomer generation (1,12,15). Furthermore, SUD prevalence in younger Americans is rising as a consequence of the opioid epidemic (34,35) and in older adults as the baby boomer generation ages (11,36). Changes in the age distribution can influence treatment and intervention programs as well as public health response, given the inherent differences in the psychosocial and medical needs of these different age groups. Our data suggest that healthcare delivery for this complex population needs to cater to both ends of the age spectrum to address the evolving needs of this heterogeneous cohort.

The sex make-up of those with comorbid CLD-SUD hospitalizations is also shifting over time. Women have experienced greater increases in comorbid admissions over time compared with men. This trend is notable because sex can affect outcomes in SUD and CLD. Women with SUD are more likely to have comorbid psychiatric disease and less likely to use alcohol SUD treatment and respond differently to treatment (37,38). It is well-established that women are more likely to develop alcoholic CLD after lower amounts of alcohol consumption and have experienced faster growths in alcohol-related mortality (11,39). Similarly, in NAFLD, women are more likely to have advanced liver disease once NAFLD is established (40). Because the rates of CLD and SUD grow in women, medical and public professionals will need to improve efforts to target the specific needs of women throughout the natural history of comorbid CLD-SUD.

The distribution of race/ethnicity is also changing over time in comorbid hospitalizations. Our data show a large growth in the number of Native Americans and non-Hispanic whites being hospitalized for CLD-SUD. The highest rates of growth in CLD in Native Americans are seen in young adults, warranting urgent public policies to support the diagnosis and treatment of CLD and SUD to reduce premature mortality predicted in this population (1,11,20,21).

Finally, those from lower income zip codes and those who have public insurance were disproportionately represented in the comorbid CLD-SUD group and were observed to increase over time. These findings mirror the trend observed in hospitalized patients for CLD-only and SUD-only groups. These trends are important to consider because previous studies have shown that the impacts of public policies, such as taxation and raising the cost of alcoholic beverages, have more pronounced impacts on those with lower socioeconomic status (41).

Our study has identified multiple risk factors that have important implications for the prevention and management of CLD and SUD. For example, in those with HCV, diagnosing and treating SUDs have been shown to improve HCV treatment uptake and efficacy with improved liver disease outcomes in those receiving integrated care (42–44). Alternatively, a higher risk of infections is noted in individuals with an SUD because of immunosenescence induced by chronic HCV infection (45). In alcohol, cirrhosis-related mortality decreased when the number of SUD treatments increased (46,47). Collectively, our study supports the imperative for providers to consider comorbidity with SUD (and vice versa) when developing a management plan. In fact, despite the approval of highly effective therapy in 2014, we show that the rates of HCV-related CLD hospitalizations have not significantly declined. This trend highlights the challenges experienced by individuals with comorbid SUD and HCV (48,49). Similarly, those with alcoholic liver disease are more likely to have comorbid psychiatric and nonalcohol-related SUDs and experience fragmented care leading to poor outcomes (5,16). Barriers to treatment encompass system-level, provider-level, and individual-level factors in this at-risk population. Our study highlights the need for healthcare delivery interventions that address all types of SUDs for anyone with CLD regardless of etiology. Beyond awareness and education about comorbid CLD-SUD, gastroenterologists and hepatologists serving this complex population need healthcare delivery systems in place to support truly integrated care. These systems can provide early identification of comorbid CLD-SUD through screening and then extend the treatment of CLD to include comorbid SUD when present through integrated care delivery models (5,38,50,51).

Our analysis has other important strengths. By using nationwide data, our conclusions are supported by a large sample size and generalizable to the entire US population. In addition, ours is the first study to capture longitudinal trends of comorbid CLD-SUD at the national level using more contemporary data. Finally, measuring the burden of comorbid CLD-SUD and understanding the demographic shifts within the hospitalized population offer important insight into treatment and management because each hospitalization may represent a unique access point for SUD and CLD treatment within the shared natural history of both diseases.

Our study is not without limitations. Our observations are limited to those who are hospitalized for CLD or SUD and may underestimate the true occurrence of each condition by not considering less severe manifestations that do not result in hospitalizations and the inability to capture admissions to alcoholism/chemical dependency treatment centers. As with other comorbid conditions, SUD may also be undercoded in those with more severe liver disease (6,52). Because of the retrospective observational data set, our findings can only be interpreted as associations, not as cause-and-effect. Despite these limitations, our analysis of NIS data captured up to 15 diagnostic codes ensuring that the most important diagnoses and procedures are cataloged. In addition, as our study was focused on the changing trends in demographic characteristics of those with comorbid CLD-SUD, future studies will be needed to provide an in-depth investigation of our observation that those with comorbid CLD-SUD have a higher age-adjusted inpatient mortality rate compared with the CLD only group. A future study using a more granular data set is needed to explore the drivers of higher mortality, such as differences in the severity of liver disease, prevalence of medical and psychiatric comorbidities, and shared biological mechanisms that may compound poor outcomes when the diseases coexist (53,54).

In conclusion, in this comprehensive analysis of 13 years of US hospitalizations, we show that comorbid CLD and SUD hospitalizations are increasing over time and lead to higher inpatient mortality than CLD alone. Although rates of alcoholic SUD and CLD continue to increase, drug use disorders are a growing proportion of SUDs affecting those with CLD. In parallel, NAFLD is a rapidly growing contributor to comorbid CLD-SUD hospitalizations. We further characterize the changing demographics of these hospitalizations, thus providing a contemporary yet inclusive look at comorbid CLD-SUD hospitalizations. Through understanding these trends, providers can more precisely deliver targeted interventions to improve the poor outcomes suffered by the growing population with both CLD and SUD. These data also call for future studies looking into shared mechanisms of NAFLD and SUD, which are increasingly affecting young, female, and ethnically diverse portions of the US population.

## CONFLICTS OF INTEREST

**Guarantor of the article:** Archita P. Desai, MD.

**Specific author contributions:** A.P.D., M.G., and N.M.: study concept and design, and data analysis. A.P.D., M.G., and L.N.: article preparation. All authors: critical review of the article. The funder was not involved in the design and conduct of the study; collection, management, analysis, and interpretation of the data; preparation, review, or approval of the article; or decision to submit the article for publication.

**Financial support:** A.P.D. was funded by National Institute of Diabetes and Digestive and Kidney Diseases of the National Institutes of Health under the award number K23DK123408. The content is solely the responsibility of the authors and does not necessarily represent the official views of the National Institutes of Health.

**Potential competing interests:** N.C. has ongoing paid consulting activities (or had in preceding 12 months) with NuSirt, Abbvie, Afimmune (DS Biopharma), Allergan (Tobira), Madrigal, Siemens, Foresite, Galectin, Zydus, and La Jolla; these consulting activities are generally in the areas of nonalcoholic fatty liver disease and drug hepatotoxicity; N.C. receives research grant support from Exact Sciences, Intercept, and Galectin Therapeutics where his institution receives the funding; and over the last decade N.C. has served as a paid consultant to more than 35 pharmaceutical companies, and these outside activities have regularly been disclosed to his institutional authorities. Remaining authors have no disclosures to report.

**Study Highlights**

**WHAT IS KNOWN**

✓ Chronic liver disease (CLD) and substance use disorders (SUDs) are increasingly prevalent in the United States and often coexist.
✓ The contemporary trends and outcomes in hospitalizations for comorbid CLD and SUD are unknown.

**WHAT IS NEW HERE**

✓ Hospitalizations with comorbid CLD and SUD result in higher age-adjusted inpatient mortality.
✓ Over time, comorbid hospitalizations are growing, and the demographics of the comorbid hospitalizations are shifting.
✓ Over time, drug use disorders and nonalcoholic fatty liver disease are increasingly prevalent in those with comorbid CLD-SUD.

**TRANSLATIONAL IMPACT**

✓ These data guide interventions needed in this high-risk group by highlighting the increasing burden of comorbid CLD-SUD hospitalizations, confirming continued poor outcomes, and identifying the contemporary characteristics of these at-risk individuals.

## Supplementary Material

SUPPLEMENTARY MATERIAL
