# Supplementary material for: Contemporary Trends in Hospitalizations for Comorbid Chronic Liver Disease and Substance Use Disorders
Source: Clin Transl Gastroenterol. 2021 Jun 18;12(6):e00372. doi: 10.14309/ctg.0000000000000372 (PMC8216677; doi:10.14309/ctg.0000000000000372)
Supplement: SUPPLEMENTARY MATERIAL [file ct9-12-e00372-s001.pdf]

SUPPLEMENTAL TABLES & FIGURE LEGENDS

Supplementary Table 1. *ICD-9* and *ICD-10* Codes based definitions of clinical variables used in data analysis.

Supplementary Table 2. Prevalence of SUD type in those with SUD alone vs Comorbid CLD-SUD (based on 2016-2017 only).

Abbreviations: CLD, chronic liver disease; *ICD-9/ICD-10*, *International Classification of Diseases, Ninth/Tenth Revision*; SUD, substance use disorder

Supplementary Table 1. *ICD-9* and *ICD-10* Codes based definitions of clinical variables

Variable	*ICD-9* codes*	*ICD-10* codes*
**Liver Disease Diagnoses**		
Hepatitis C	070.44, 070.54, 070.70, 070.71, V02.62	B18.2, B192.0, B192.1, Z22.52
Alcohol	571.0, 571.1, 571.2, 571.3	K70.x
NASH	571.5, 571.40, 571.41, 571.49<br>AND<br>Code for Diabetes or Obesity	K73.x, K74.60, K74.69<br>AND<br>Code for Diabetes or Obesity
Other	273.4, 275.1, 275.01, 275.03, 571.42, 571.6, 571.6, 571.6, 576.1, 070.49, 070.59, 07.06, 07.09, 573.3, 571.40, 571.41, 571.49, 571.49, 571.9, 573.9	E88.01, E83.00, E83.01, E83.09, E83.110, E83.118,<br>K75.4, K74.3, K74.4, K74.5, K83.01, K83.09,<br>B17.2, B17.8, B19.0, B19.9, K71.6, K73.9, K73.0, K73.2, K73.8, K74.1, K76.89, K76.9
Cirrhosis	571.2, 571.5, 571.6<br>AND<br>572.3, 789.59, 572.2, 456.0, 456.20, 578.0, 578.1, 578.9, 572.4, 789.5x but not 789.51	K74.x, K70.3, K70.2, K76.1, K71.7<br>AND<br>K76.6, R18.8, K70.31, K70.11, K71.51, K70.41, K72.01, K72.11, K72.91, B19.0, B19.11, B19.21, I85.01, I85.11, K92.0, K92.1, K92.2, K76.7
**Substance Use Diagnoses**		
Alcohol use disorder	291, 303, 305.0	F10
Drug use disorder	292, 304, 305.2, 305.3, 305.4, 305.5, 305.6, 305.7, 305.8, 305.9	F11, F12, F13, F14, F15, F16, F18, F19

* Condition considered present if listed codes present in diagnosis and procedure codes, levels 1-15 for each. Comma represents “or”.

Supplementary Table 2. Prevalence of SUD type in those with SUD alone vs Comorbid CLD-SUD (based on 2016-2017 only).

Type of Disease	SUD only N=5,083,912 (%)	Comorbid CLD-SUD N=1,249,174 (%)
Alcohol (F10)	48.7%	69.0%
Cannabis (F12)	26.5%	11.3%
Opioids (F11)	22.6%	24.0%
Cocaine (F14)	12.4%	10.4%
Stimulants (F15)	10.1%	7.5%
Sedatives, Hypnotics, Anxiolytics (F13)	4.1%	2.8%
Hallucinogens (F16)	0.6%	0.2%
Inhalants (F18)	0.1%	0.1%
Other Psychoactive Substances (F19)	8.1%	7.1%

*Break down only available for 2016-2017 when *ICD-10* available

Supplementary Figure

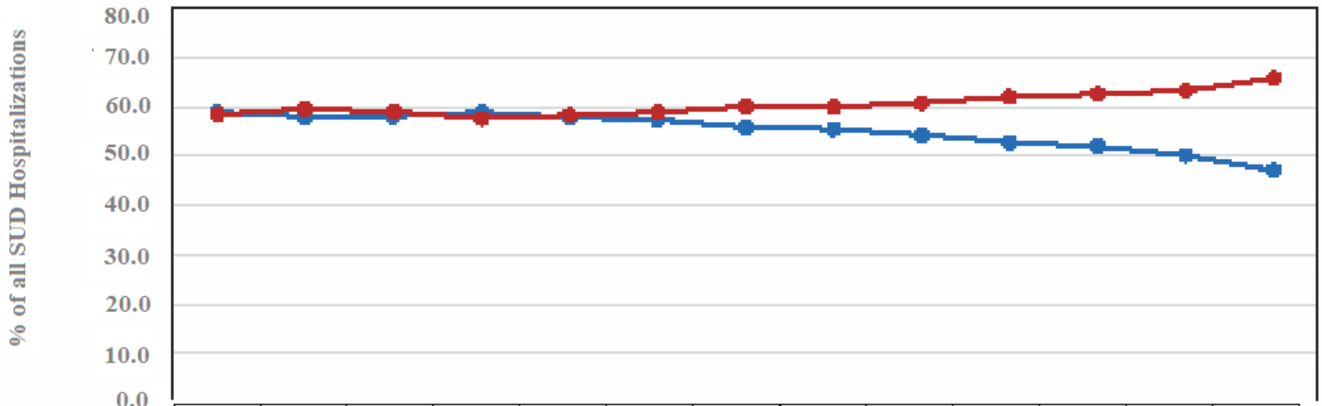

	2005	2006	2007	2008	2009	2010	2011	2012	2013	2014	2015	2016	2017
Alcohol disorder	58.9%	58.0%	58.1%	58.9%	57.9%	57.4%	55.9%	55.5%	54.4%	52.8%	52.0%	50.2%	47.1%
Drugs	58.4%	59.6%	59.0%	57.7%	58.3%	58.9%	60.1%	60.0%	60.9%	62.6%	62.6%	63.3%	65.9%

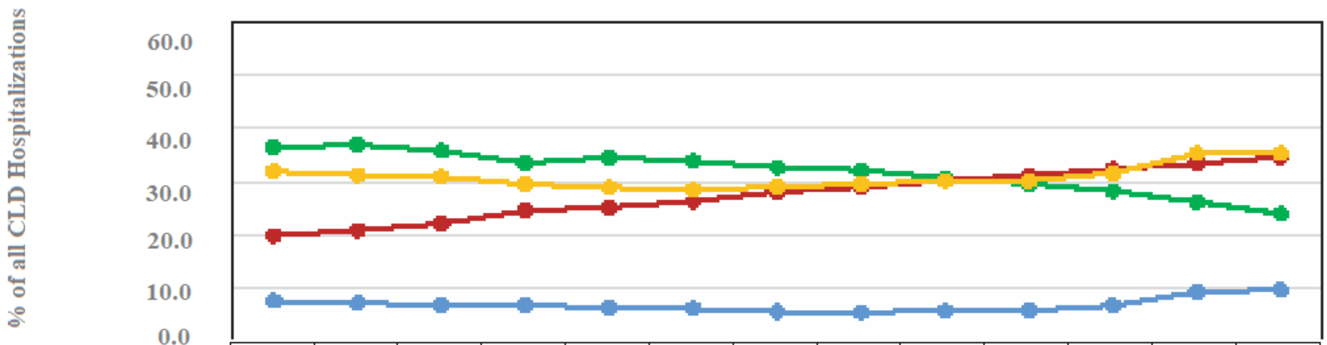

	2005	2006	2007	2008	2009	2010	2011	2012	2013	2014	2015	2016	2017
Alcoholic liver disease	7.6%	7.2%	6.9%	6.7%	6.3%	6.1%	5.65%	5.5%	5.6%	5.8%	6.7%	9.3%	9.7%
Hepatitis C	36.4%	37.0%	35.9%	33.5%	34.6%	33.9%	32.9%	32.2%	30.7%	29.6%	29.2%	26.2%	24.0%
NAFLD	19.9%	20.9%	22.2%	24.5%	25.2%	26.3%	29.0%	29.0%	30.2%	31.2%	32.4%	33.4%	34.6%
Cirrhosis	31.9%	31.2%	30.9%	29.6%	29.4%	29.%	29.%	29.5%	30.2%	30.2%	31.6%	35.3%	35.3%

Supplementary Figure 1. Trends in hospitalizations by liver disease and substance use type over the study period. (A) Percent of all SUD hospitalizations with alcohol and drug use disorders over the study period. (B) Percent of all CLD hospitalizations with alcoholic liver disease, hepatitis C, NAFLD and cirrhosis over the study period.

Abbreviations: CLD, chronic liver disease; NAFLD, non-alcoholic fatty liver disease; SUD, substance use disorder.
